# The Prevalence of Sexual Misconduct in US Medical Education: Examining the Intersecting Vulnerabilities of Gender and Sexual Orientation

**DOI:** 10.7759/cureus.63681

**Published:** 2024-07-02

**Authors:** Daryl O Traylor, Eboni Anderson, Cooper K Allenbrand, Taranjit Kaur, Harnoor Gill, Harsimran Singh

**Affiliations:** 1 Public Health, A.T. Still University School of Osteopathic Medicine, Mesa, USA; 2 Basic Sciences, University of the Incarnate Word School of Osteopathic Medicine, San Antonio, USA; 3 Basic Sciences, William Carey University College of Osteopathic Medicine, Hattiesburg, USA; 4 Department of Biology, University of California, Berkeley, Berkeley, USA

**Keywords:** allopathic, osteopathic, united states, medical education, sexual misconduct

## Abstract

This study explores the prevalence, characteristics, and correlates of sexual harassment and nonconsensual sexual contact among medical students in the United States (US). The study aims to understand the association between gender, sexual orientation, and these incidents within the context of undergraduate medical education in the US. Employing a cross-sectional approach, this study collected primary data from 23,124 medical students across various US allopathic and osteopathic medical schools. After the data were cleaned, 245 (1% of the targeted population) respondents were included in the final analysis. The focus was on the prevalence and characteristics of sexual harassment and nonconsensual sexual contact and the association of gender and sexual orientation with these experiences. The findings revealed that 12.2% (n = 30) of the respondents experienced nonconsensual sexual contact, with other medical students being the most common perpetrators. A significant association was found between gender, sexual orientation, and the occurrence of unwanted sexual contact, indicating a disproportionate impact on non-heterosexual individuals and females. The study underscores the prevalence of sexual harassment and nonconsensual sexual contact within the US undergraduate medical education, highlighting disparities based on gender and sexual orientation. These results call for the implementation of policies and programs to address sexual misconduct in medical schools. The study elucidates the need for an understanding of the impact of sexual misconduct on students attending both allopathic (MD) and osteopathic (DO) medical programs.

## Introduction

Sexual misconduct, harassment, and sexual violence are critical issues facing United States (US) undergraduate medical education [1,2]. These experiences can have profound impacts on the mental health, academic performance, and professional trajectories of medical students [3-5]. Despite growing awareness, there remains a significant gap in understanding the prevalence, characteristics, and impacts of these experiences among medical students. In this study, "nonconsensual sexual contact" refers to any unwanted sexual touching; "sexual harassment" involves unwelcome sexual advances, requests for sexual favors, and other verbal or physical harassment of a sexual nature; and "sexual violence" encompasses acts of sexual coercion, assault, and rape [6-8]. Undergraduate medical education in the US context is defined as the training pathway leading to the Doctor of Medicine (MD) or Doctor of Osteopathy (DO) degrees, focusing on the pre-residency phase of medical training in the US.

There is a growing awareness of sexual harassment and sexual violence in higher education [9]. Movements and campaigns, both national and global, have shed light on the prevalence and severity of these issues [10]. However, despite this increased attention, significant challenges persist. The medical education hierarchy, characterized by distinct power dynamics between students, residents, and faculty, may contribute to environments where sexual misconduct, harassment, and violence are more likely to occur. This study examines these dynamics as potential factors in reported incidents of sexual misconduct [11-13]. This environment can lead to underreporting and a lack of accountability, allowing such behaviors to continue unchecked.

While there is an increasing body of literature on sexual misconduct, harassment, and sexual violence in higher education, there remains a substantial gap in understanding these phenomena specifically within medical schools [12-15]. Medical students face unique pressures, including intense academic demands, clinical rotations, and patient care responsibilities. These factors can influence how sexual harassment and sexual violence manifest and are addressed in medical educational settings. Moreover, the potential impact of these experiences on future healthcare professionals, who are tasked with patient care and ethical decision-making, underscores the need for a deeper understanding in this area [16-18].

The implications of sexual misconduct, harassment, and sexual violence in medical education extend beyond immediate distress. They can have lasting effects on mental health, leading to anxiety, depression, and burnout [16,17]. Such outcomes not only affect individual students but can also have broader implications for the quality of patient care and the overall healthcare system. Prior studies, such as the McClain et al. (2020) study, have highlighted that sexual and gender minority (SGM) and female students are particularly vulnerable to these adverse experiences [17]. SGM medical students face significant stigma and discrimination, leading to chronic stress and lower social support [17]. Attrition rates are notably higher among these groups due to the compounded stress and trauma associated with such incidents. Furthermore, these experiences can disrupt academic progress and professional development, potentially leading to a decrease in the diversity of the medical profession and a loss of talented individuals from the field [18].

Comprehensive research into the prevalence, characteristics, and impacts of sexual harassment and sexual violence among medical students is crucial. Such research can inform the development of targeted policies and interventions to prevent these incidents and support affected students. Understanding the scope and nature of these issues within medical schools is essential for creating safe and inclusive learning environments, promoting student well-being, and ensuring the ethical and professional development of future medical practitioners.

Objective of the study

This national study aims to explore the prevalence and characteristics of sexual harassment and nonconsensual or unwanted sexual contact among medical students. It seeks to understand the association between gender, sexual orientation, and the occurrence of these experiences among medical students.

## Materials and methods

Study design

This cross-sectional study, conducted at the University of the Incarnate Word School of Osteopathic Medicine, San Antonio, Texas, employed a survey-based approach to assess the prevalence and characteristics of nonconsensual or unwanted sexual contact and sexual harassment among medical students. The survey was distributed to a target population of 23,124 medical students across various US allopathic (MD) and osteopathic (DO) medical schools. Data collection occurred from August 2, 2023, to November 30, 2023, utilizing Facebook, Twitter, Instagram, and LinkedIn for recruitment. Recruitment postings were made bi-weekly to relevant groups and forums to maximize reach. After the data were cleaned, the final sample consisted of 245 respondents who provided complete or partially complete responses. The missing responses referred to in this context include various types of incomplete responses from the survey and were not specifically related to questions about sexual harassment and non-consensual sexual contact.

Research questions and analysis

The research questions are as follows: 1) What are the prevalence and characteristics of nonconsensual or unwanted sexual contact among medical students? 2) Are there differences in the occurrences of unwanted sexual contact and sexual harassment between students attending MD-granting and DO-granting medical schools? 3) What are the prevalence and characteristics of sexual harassment among medical students? 4) What is the association between gender, sexual orientation, and unwanted sexual contact? 5) Is there a difference in the occurrence of sexual harassment based on the gender and sexual orientation of the individuals involved?

Sample size calculation

The sample size needed for prevalence estimates was determined based on confidence level and margin of error. Assuming the study sample will be a relatively small fraction of the population, approximately 384 participants would be needed to compute prevalence estimates with a 95% level of confidence and a 5% margin of error.

G*Power software (developed by Franz Faul et al., 2020, Kiel University, Germany) was used to determine the sample sizes needed for the proposed inferential tests to detect medium-sized effects at an alpha level of 0.05 and a power level of 0.80. For a chi-square test with up to five degrees of freedom, the minimum sample size needed was 143. For a binary logistic regression coefficient, a minimum sample size of 177 respondents who experienced non-consensual sexual contact was needed. For a Mann-Whitney test, the minimum sample size needed was 134.

Participant recruitment

The participants for this study were recruited through a targeted approach on social media platforms, utilizing groups and communities frequented by medical students at various stages of their education. The survey instrument was distributed via Qualtrics, a widely recognized survey tool, ensuring ease of access and confidentiality. The specific platforms used for dissemination included Facebook, X (formerly Twitter), Instagram, and LinkedIn, each chosen for their popularity and extensive use among medical students. This strategy was designed to reach a diverse sample of medical students, facilitating the collection of data from a broad spectrum of experiences and perspectives in the field of medical education.

Instrument

In this study, we adapted the Revised Campus Climate Survey from the Administrator-Researcher Campus Climate Collaborative (ARC3) for medical education settings. The original ARC3 survey, tested across three universities with 909 students and achieving an 85% completion rate, encompasses 19 modules on Title IX violations and related issues [19]. Our adaptation involved customizing survey items for the undergraduate medical school environment, ensuring relevance while maintaining the survey's primary goals. Importantly, the psychometric analysis of the original ARC3 survey demonstrated high internal reliability (18 out of 25 scales with coefficients of 0.80 or above), and the associations among different modules were consistent with established research [19]. This high internal reliability made the survey an effective tool for capturing the unique experiences of medical students. The adapted ARC3 instrument was not pilot-tested, but it was implemented directly without preliminary evaluation.

Ethical considerations

Ethical approval was obtained from the University of the Incarnate Word Institutional Review Board (IRB approval #2023-1425-EXP-v4). The participants provided informed consent, and measures were taken to ensure the anonymity and confidentiality of the data. The data collected were primary survey data, and no Internet Protocol (IP) addresses or other identifying information were collected or recorded. All data are stored on an encrypted hard drive, and analyses were conducted in compliance with ethical standards for research involving human subjects.

## Results

The demographic characteristics of the sample are presented in Table 1. Among the 231 respondents who provided their age, their ages ranged from 21 to 52 years (M = 26.54, SD = 4.72). Most respondents identified as female (n = 140, 57.1%). Most respondents indicated that they were not Hispanic or Latino (n = 206, 84.1%), and most respondents identified their race as White (n = 129, 52.7%). Approximately three-quarters of the respondents identified as heterosexual (n = 187, 76.3%). Most respondents reported being in their second year of medical school (n = 99, 40.4%), and most respondents attended an osteopathic medical school (n = 131, 53.5%).

**Table 1:** Sample characteristics

Variable	Frequency	Percent
Gender identity		
Male	88	35.9
Female	140	57.1
Transgender male	4	1.6
Transgender female	4	1.6
Genderqueer/gender‐nonconforming	9	3.7
Ethnicity		
Hispanic or Latino	36	14.7
Not Hispanic or Latino	206	84.1
Missing/did not answer	3	1.2
Race		
American Indian or Alaska Native	5	2.0
Asian	53	21.6
Black or African American	61	24.9
Native Hawaiian or other Pacific Islander	7	2.9
White	129	52.7
Sexual orientation		
Bisexual	29	11.8
Gay	13	5.3
Heterosexual	187	76.3
Lesbian	6	2.4
Questioning	3	1.2
Other	6	2.4
Missing/did not answer	1	0.4
What is your current status in medical school?		
1st year	54	22.0
2nd year	99	40.4
3rd year	46	18.8
4th year	46	18.8
What type of medical school do you attend?		
Allopathic (MD granting)	113	46.1
Osteopathic (DO granting)	131	53.5
Missing/did not answer	1	0.4

Research questions

What Is the Prevalence and Characteristics of Nonconsensual or Unwanted Sexual Contact Among Medical Students?

To answer RQ1, frequencies and percentages were computed for the responses to the questions pertaining to nonconsensual or unwanted sexual contact. Included were questions about whether the respondent has experienced unwanted sexual contact, the nature of the incident, characteristics of the perpetrator, and events afterward (i.e., filing a complaint). Margins of error (MOE) for prevalence were calculated based on a sample size of 245, a population size of 23,124, and a confidence level of 95%.

Table 2 displays the frequencies and percentages for the prevalence and characteristics of nonconsensual or unwanted sexual contact. Approximately 12.2% of the respondents (n = 30, MOE ± 4.1%) reported that someone had sexual contact with them by using physical force or threats of harm. Approximately 10.6% of the respondents (n = 26, MOE ± 3.8%) reported that someone had attempted but not succeeded in having sexual contact with them by using or threatening to use physical force. Approximately 6.5% of the respondents (n = 16, MOE ± 3.1%) reported that someone had sexual contact with them when they were unable to provide consent or stop what was happening, and 5.7% of the respondents (n = 14, MOE ± 2.9%) suspected but were not certain that this had happened to them.

**Table 2:** Prevalence and characteristics of nonconsensual or unwanted sexual contact among medical students since 2020

Question	Frequency	Percent
Has anyone had sexual contact with you by using physical force or threatening to physically harm you?		
Yes	30	12.2
No	205	83.7
Missing/Other	10	4.1
Has anyone attempted but not succeeded in having sexual contact with you by using or threatening to use physical force against you?		
Yes	26	10.6
No	208	84.9
Missing/Other	11	4.5
Since 2020, has someone had sexual contact with you when you were unable to provide consent or stop what was happening because you were passed out, drugged, drunk, incapacitated, or asleep?		
Yes	16	6.5
No	218	89.0
Missing/Other	11	4.5
Have you suspected that someone has had sexual contact with you when you were unable to provide consent or stop what was happening because you were passed out, drugged, drunk, incapacitated, or asleep?		
Yes	14	5.7
No	220	89.8
Missing/Other	11	4.5
When the person had sexual contact with you when you were unable to provide consent or stop what was happening because you were passed out, drugged, drunk, incapacitated, or asleep, which of the following happened?		
Forced touching of a sexual nature	12	4.9
Oral sex	9	3.7
Sexual intercourse	17	6.9
Anal sex	6	2.4
Sexual penetration with a finger or object	11	4.5
Who did the unwanted behavior involve?		
Another student or peer	38	15.5
Staff member at your medical school	2	0.8
A resident physician	2	0.8
An attending physician	1	0.4
Someone else affiliated with your medical school or hospital/clinic	1	0.4
Other	17	6.9
What was the gender of the individual who did this to you?		
Male	44	18
Female	4	1.6
Non-binary	1	0.4
Not sure	7	2.9
Missing/Other	189	77.1
Where did the incident occur?		
Off campus	40	16.3
On campus	5	2.0
A clinical site	2	0.8
Other location	13	5.3
Did you initiate the university's process to file a sexual assault complaint?		
Yes	25	10.2
No	48	19.6
Missing/Other	172	70.2
If yes, did university's formal procedures help you deal with the problem?		
Didn't help me at all	6	2.4
Helped me a little	12	4.9
Helped, but could have helped more	5	2.0
Helped me a lot	5	2.0
Missing/Other	217	88.6
If you did not tell anyone, why?		
Ashamed/embarrassed	16	6.5
Fear of retribution from the person who did it	14	5.7
Fear of not being believed by my school	10	4.1
Didn't know my school's reporting procedure	4	1.6
I did not feel that my campus leadership would solve the problem	13	5.3
Feared that reporting the incident would impact my academics	13	5.3
Feared that reporting the incident would impact my chances of earning the residency that I want	10	4.1
Didn’t want others to worry about me	12	4.9
Didn't think that my school would do anything about the report	7	2.9
Wanted to forget it happened	15	6.1
Didn’t want others to worry about me	7	2.9

Seventeen respondents (6.9% of the total sample) reported that sexual intercourse had occurred when they were unable to provide consent or stop what was happening. The most reported perpetrator of unwanted behavior was another student or peer (n = 38, 15.5%), and the most reported gender of the perpetrator was male (n = 44, 18.0%). The largest proportion of respondents reported that unwanted behavior occurred off campus (n = 40, 16.3%). Twenty-five respondents (10.2%) indicated that they had initiated the university's process to file a sexual assault complaint, and 48 respondents (19.6%) had not. Twenty-two respondents (9.0%) indicated that the university's formal procedures helped compared to six (2.4%) who indicated that the formal procedures did not help at all. The most reported reasons why respondents did not disclose the incidents to others were shame/embarrassment (n = 16, 6.5%), desire to forget the incident (n = 15, 6.1%), and fear of retribution from the person who did it (n = 14, 5.7%).

How Do the Occurrences of Unwanted Sexual Contact and Sexual Harassment Differ Between Students Attending MD-Granting and DO-Granting Medical Schools?

Crosstabulations and chi-square tests of independence were conducted to determine if there were differences between MD and DO students in the occurrence of unwanted sexual contact and sexual harassment. The independent variable in this analysis was the type of medical school attended (MD granting or DO granting). The dependent variables were the survey questions asking about the occurrence of unwanted sexual contact and sexual harassment.

Table 3 displays the crosstabulation of the type of medical school and the occurrence of unwanted sexual contact and sexual harassment. There was no significant association between the type of medical school and sexual contact by force or threat of harm (χ2(1) = 1.19, p = 0.275). There was a significant association between the type of medical school and attempted sexual contact by force or threat of harm (χ2(1) = 10.51, p = 0.001). Specifically, 18% (n = 19) of the MD respondents experienced this, which was significantly more frequent than the 5% (n = 6) of the DO respondents who experienced this. There was no significant association between the type of medical school and sexual contact when the respondent was unable to provide consent or stop what was happening (χ2(1) = 1.28, p = 0.258). There was no significant association between the type of medical school and suspected sexual contact when the respondent was unable to provide consent or stop what was happening (χ2(1) = 0.00, p = 0.986). There was no significant association between the type of medical school and sexual harassment (χ2(1) = 2.57, p = 0.109).

**Table 3:** Crosstabulation of the type of medical school and unwanted sexual contact/harassment

	Type of Medical School		
Question	MD	DO	p
Has anyone had sexual contact with you by using physical force or threatening to physically harm you?			0.275
Yes	16 (15%)	13 (10%)	
No	91 (85%)	114 (90%)	
Has anyone attempted but not succeeded in having sexual contact with you by using or threatening to use physical force against you?			0.001
Yes	19 (18%)	6 (5%)	
No	87 (82%)	121 (95%)	
Since 2020, has someone had sexual contact with you when you were unable to provide consent or stop what was happening because you were passed out, drugged, drunk, incapacitated, or asleep?			0.258
Yes	9 (8%)	6 (5%)	
No	98 (92%)	120 (95%)	
Have you suspected that someone has had sexual contact with you when you were unable to provide consent or stop what was happening because you were passed out, drugged, drunk, incapacitated, or asleep?			0.986
Yes	6 (6%)	7 (6%)	
No	101 (94%)	119 (94%)	
Have you ever experienced any episodes of sexual harassment since 2020?			0.109
Yes	48 (48%)	45 (37%)	
No	53 (52%)	77 (63%)	

53 (52%)	77 (63%)

What Are the Prevalence and Characteristics of Sexual Harassment Among Medical Students?

To answer RQ3, frequencies and percentages were computed for the responses to the questions pertaining to sexual harassment. Included were questions about whether the respondent had experienced sexual harassment, the frequency and nature of the harassment, and the characteristics of the perpetrator. Table 4 displays the frequencies and percentages for the prevalence and characteristics of sexual harassment. Approximately 38.4% of the respondents (n = 94, MOE ± 6.1%) reported that they had experienced sexual harassment. Thirty respondents (12.2% of the total sample) indicated that they experienced sexual harassment only once, but 39 respondents (15.9%) reported experiencing sexual harassment more than 10 times. The most reported types of harassment respondents experienced were suggestive remarks (n = 61, 24.9%) and jokes of a sexual nature (n = 55, 22.4%). The largest proportion of respondents reported that the harassment occurred on campus (n = 62, 25.3%), and the most reported perpetrator of harassment was a student or peer (n = 68, 27.8%).

**Table 4:** Prevalence and characteristics of sexual harassment among medical students since 2020

Question	Frequency	Percent
Have you ever experienced any episodes of sexual harassment since 2020?		
Yes	94	38.4
No	130	53.1
Missing/Other	21	8.6
How often has this happened to you since 2020?		
Weekly	8	3.3
Monthly	10	4.1
Only once	30	12.2
2-4 times	39	15.9
5-10 times	9	3.7
More than 10 times	39	15.9
Missing/Other	110	44.9
What type(s) of sexual harassment have you experienced since 2020?		
Unwanted physical contact	26	10.6
Suggestive remarks	61	24.9
Jokes of a sexual nature	55	22.4
Display of sexually offensive materials in a public space	7	2.9
Unwanted comments on dress or appearance	47	19.2
Invasion of personal space	39	15.9
Staring or leering	41	16.7
Intimidating presence	30	12.2
None	124	50.6
Where did this happen to you?		
On campus	62	25.3
Hospital or clinic	40	16.3
Other	43	17.6
Who did this to you?		
Student or peer	68	27.8
Faculty member	13	5.3
Staff member on campus	7	2.9
Resident physician	16	6.5
Attending physician	16	6.5
Staff member at hospital or clinic	13	5.3
Other	25	10.2

What Are the Associations Between Gender, Sexual Orientation, and Unwanted Sexual Contact?

Table 5 displays the crosstabulation of respondent gender and the occurrence of unwanted sexual contact. There was a significant association between gender and sexual contact by force or threat of harm (χ2(2) = 7.28, p = 0.026). Specifically, 7% (n = 6) of the male respondents experienced this, which was significantly less frequent than the 29% (n = 5) of other gender respondents who experienced this. There was no significant association between gender and attempted sexual contact by force or threat of harm (χ2(2) = 3.79, p = 0.151). There was no significant association between gender and sexual contact when the respondent was unable to provide consent or stop what was happening (χ2(2) = 0.74, p = 0.689). There was no significant association between gender and suspected sexual contact when the respondent was unable to provide consent or stop what was happening (χ2(2) = 5.30, p = 0.071).

**Table 5:** Crosstabulation of gender and unwanted sexual contact

	Gender n (%)			
Question	Male	Female	Other	p
Has anyone had sexual contact with you by using physical force or threatening to physically harm you?				0.026
Yes	6 (7%)	19 (15%)	5 (29%)	
No	81 (93%)	112 (85%)	12 (71%)	
Has anyone attempted but not succeeded in having sexual contact with you by using or threatening to use physical force against you?				0.151
Yes	11 (13%)	11 (8%)	4 (24%)	
No	76 (87%)	119 (92%)	13 (76%)	
Since 2020, has someone had sexual contact with you when you were unable to provide consent or stop what was happening because you were passed out, drugged, drunk, incapacitated, or asleep?				0.689
Yes	6 (7%)	8 (6%)	2 (12%)	
No	81 (93%)	122 (94%)	15 (88%)	
Have you suspected that someone has had sexual contact with you when you were unable to provide consent or stop what was happening because you were passed out, drugged, drunk, incapacitated, or asleep?				0.071
Yes	6 (7%)	5 (4%)	3 (18%)	
No	81 (93%)	125 (96%)	14 (82%)	

Table 6 displays the crosstabulation of respondent sexual orientation and the occurrence of unwanted sexual contact. There was a significant association between sexual orientation and sexual contact by force or threat of harm (Fisher’s exact p < 0.001). Specifically, 22% (n = 6) of the bisexual respondents and 39% (n = 7) of the gay/lesbian respondents experienced this, which was significantly more frequent than the 8% (n = 15) of heterosexual respondents who experienced this. There was a significant association between sexual orientation and attempted sexual contact by force or threat of harm (Fisher’s exact p < 0.001). Specifically, 26% (n = 7) of the bisexual respondents and 50% (n = 9) of the gay/lesbian respondents experienced this, which was significantly more frequent than the 5% (n = 9) of the heterosexual respondents who experienced this. There was a significant association between sexual orientation and sexual contact when the respondent was unable to provide consent or stop what was happening (Fisher’s exact p = 0.030). Specifically, 15% (n = 4) of the bisexual respondents and 17% (n = 3) of the gay/lesbian respondents experienced this, which was significantly more frequent than the 4% (n = 8) of heterosexual respondents who experienced this. There was a significant association between sexual orientation and suspected sexual contact when the respondent was unable to provide consent or stop what was happening (Fisher’s exact p = 0.007). Specifically, 17% (n = 3) of the gay/lesbian respondents and 22% (n = 2) of other orientation respondents experienced this, which was significantly more frequent than the 3% (n = 6) of heterosexual respondents who experienced this.

**Table 6:** Crosstabulation of sexual orientation and unwanted sexual contact

Question	Bisexual	Gay/Lesbian	Heterosexual	Other	p
Has anyone had sexual contact with you by using physical force or threatening to physically harm you?					<0.001
Yes	6 (22%)	7 (39%)	15 (8%)	2 (22%)	
No	21 (78%)	11 (61%)	165 (92%)	7 (78%)	
Has anyone attempted but not succeeded in having sexual contact with you by using or threatening to use physical force against you?					< 0.001
Yes	7 (26%)	9 (50%)	9 (5%)	1 (11%)	
No	20 (74%)	9 (50%)	170 (95%)	8 (89%)	
Since 2020, has someone had sexual contact with you when you were unable to provide consent or stop what was happening because you were passed out, drugged, drunk, incapacitated, or asleep?					0.030
Yes	4 (15%)	3 (17%)	8 (4%)	1 (11%)	
No	23 (85%)	15 (83%)	171 (96%)	8 (89%)	
Have you suspected that someone has had sexual contact with you when you were unable to provide consent or stop what was happening because you were passed out, drugged, drunk, incapacitated, or asleep?					0.007
Yes	3 (11%)	3 (17%)	6 (3%)	2 (22%)	
No	24 (89%)	15 (83%)	173 (97%)	7 (78%)	

Is There a Difference in the Occurrence of Sexual Harassment Based on the Gender and Sexual Orientation of the Individuals Involved?

To answer RQ5, crosstabulations and chi-square tests of independence were conducted to determine if there are associations between gender, sexual orientation, and the occurrence of sexual harassment. The independent variables in this analysis were respondent gender and respondent sexual orientation. The dependent variable was the survey question asking about the occurrence of sexual harassment. If the cell sizes were not adequate for the chi-square test, Fisher’s exact test was computed.

Table 7 displays the crosstabulation of respondent gender and the occurrence of sexual harassment. There was a significant association between gender and sexual harassment (χ2(2) = 22.10, p < 0.001). Specifically, 23% of the male respondents experienced sexual harassment, which was significantly less frequent than the 51% of females and 71% of other gender respondents who experienced sexual harassment.

**Table 7:** Crosstabulation of gender and sexual harassment

	Gender n (%)			
Question	Male	Female	Other	p
Have you ever experienced any episodes of sexual harassment since 2020?				<0.001
Yes	19 (23%)	63 (51%)	12 (71%)	
No	64 (77%)	61 (49%)	5 (29%)	

Table 8 displays the crosstabulation of respondent sexual orientation and the occurrence of sexual harassment. There was a significant association between sexual orientation and sexual harassment (Fisher’s exact p < 0.001). Specifically, 81% of the bisexual respondents experienced sexual harassment, which was significantly more frequent than the 50% of gay/lesbian respondents, 35% of the heterosexual respondents, and 37.5% of other orientation respondents who experienced sexual harassment.

**Table 8:** Crosstabulation of sexual orientation and sexual harassment

Question	Bisexual	Gay/lesbian	Heterosexual	Other	p
Have you ever experienced any episodes of sexual harassment since 2020?					<0.001
Yes	22 (81%)	9 (50%)	60 (35%)	3 (37.5%)	
No	5 (19%)	9 (50%)	110 (65%)	5 (62.5%)	

## Discussion

This study highlights the significant prevalence of nonconsensual or unwanted sexual contact and sexual harassment among medical students. Notably, a substantial proportion of respondents reported experiences of sexual contact through physical force or when unable to consent, with peers being the most common perpetrators. The findings also underscore the pervasive nature of sexual harassment faced by participants, often manifesting as suggestive remarks and jokes of a sexual nature.

The data further revealed a concerning association between gender and sexual orientation with the occurrence of unwanted sexual contact. Non-heterosexual students and those identifying as females reported disproportionately higher rates of such experiences. This underscores the intersectional vulnerabilities that females and non-heterosexual medical students face in the context of sexual misconduct in medical education.

Historically, medical education has been characterized by hierarchical structures that may contribute to the perpetuation of harassment and abuse [10-13,17]. Previous studies have indicated varying prevalence rates of sexual harassment and sexual violence in medical schools, with some reports suggesting that a significant proportion of medical students experience these issues during their training [13,16,18,20-22]. These experiences not only have immediate psychological and physical impacts but also long-term consequences on professional development and career trajectories.

Impact on gender and sexual orientation

Gender and sexual orientation have been identified as critical factors in the experience and impact of sexual assault. Women and individuals from the LGBTQ+ community are often disproportionately affected [23-25]. This disproportionate impact raises concerns about equity and inclusivity in medical education, which are essential for fostering a diverse and competent healthcare workforce.

Institutional responses and policies

The response of medical institutions to incidents of sexual misconduct is crucial in shaping students' perceptions of safety and support. Effective policies and interventions are needed to prevent these incidents and provide support to those affected. However, there is a lack of comprehensive data on how current institutional policies and educational programs address these issues and their effectiveness [16,26-30].

Research gap and need for study

Despite the recognized importance of this issue, there is a scarcity of large-scale, comprehensive studies examining the prevalence, characteristics, and institutional responses to sexual harassment and assault in medical schools across the United States. This study aims to help address this gap by providing empirical data on these experiences among a diverse sample of medical students, thus contributing to a more profound understanding and informing policy and practice in medical education.

Significance

The investigation into sexual misconduct, harassment, and sexual violence in US undergraduate medical education is crucial for developing targeted interventions and policies to support medical students' well-being. It also contributes to the broader discourse on institutional responsibilities in preventing and addressing sexual violence in medical education settings.

While this study encompasses responses from both MD and DO students, it does not explore the possible impacts of osteopathic manipulative treatment (OMT) on experiences of non-consensual sexual misconduct and sexual harassment in DO students. It is currently unknown whether OMT training has a protective effect in these contexts. Recognizing this as a limitation, future research should aim to specifically investigate the role of OMT within these contexts.

Interpretation of findings

The study's findings reveal a concerning prevalence of nonconsensual sexual contact and sexual harassment among medical students. The reported rates of unwanted sexual contact, particularly those involving physical force or inability to consent, highlight a critical issue within medical education institutions. The predominance of peers as perpetrators suggests a need for targeted interventions within the student community.

The high incidence of sexual harassment, often manifesting as suggestive remarks and jokes, underscores the possible pervasive nature of this problem in medical school settings. These findings align with existing literature indicating that sexual harassment is a common experience in higher education, often contributing to a hostile learning environment [30,31].

The study also sheds light on the intersectional vulnerabilities faced by non-heterosexual students and those identifying with genders other than male. The significantly higher rates of unwanted sexual contact reported by these groups point to the need for more inclusive and sensitive policies and support systems within medical schools. Research consistently shows that LGBTQ non-male university students experience higher rates of sexual violence when compared to national averages [32-34]. This is particularly concerning given the potential impact on their mental and physical health [33]. The prevalence of mistreatment in medical schools is also disproportionately higher for these students [35]. 

Our findings indicate that a significant proportion of harassment perpetrators are individuals in positions of authority, aligning with our introductory emphasis on the hierarchical nature of medical education [30]. This underscores the urgent need for interventions that address power imbalances and promote a culture of accountability and respect.

Limitations

The study has a notably low response rate of approximately 1.3%, culminating in a sample size of 245 respondents. This limited response raises concerns about the generalizability of the findings to the broader medical student community. The potential for selection bias is also evident, as the respondents' decision to participate might have been influenced by specific experiences or motivations related to the study's subject matter. The potential for bias underscores the need for cautious generalization of the study’s findings.

Furthermore, the reliance on self-reported data introduces the risk of recall bias and social desirability bias. Given the sensitive nature of the survey's topics, there is a possibility that respondents might have either underreported or overreported their experiences, thereby impacting the accuracy of the reported prevalence estimates. Furthermore, the sensitive nature of the survey topics might have led to non-response bias, particularly if those who have experienced sexual harassment or violence were less inclined to participate in the survey. Such a bias could result in an underestimation of the true prevalence and impact of these experiences among medical students.

The study's cross-sectional design further constrains its scope, specifically in its limitation to establish causal relationships. It offers a temporal snapshot of the experiences without the capacity to track changes or developments over time in the medical education context.

Another notable limitation of our study is that the majority of our participants were pre-clinical students. This demographic skew means that our findings may not fully capture the experiences of clinical students, who are likely to encounter different dynamics and power differentials in their interactions, particularly within clinical settings involving residents, attending physicians, and other healthcare professionals. As such, the experiences of sexual misconduct and sexual harassment between pre-clinical and clinical students may vary significantly, and our results cannot be generalized to both groups with equal accuracy. Furthermore, our adapted ARC3 instrument did not specifically differentiate episodes of sexual violence occurring at home at the hands of a partner from instances of sexual misconduct and violence within school or clinical environments perpetrated by partners, classmates, faculty members, or staff members. This lack of differentiation may obscure the context in which these incidents occurred, potentially conflating distinct experiences of violence. We acknowledge these limitations and suggest that future research should aim to address these gaps to provide a more comprehensive understanding of sexual misconduct and harassment across different stages of medical training and environments.

An additional limitation is the study's primary focus on gender and sexual orientation, while other crucial demographic variables such as race, ethnicity, age, and academic year were not thoroughly examined. This oversight restricts a comprehensive understanding of the intersectional factors that might influence experiences of sexual harassment and violence.

Finally, the utilization of social media platforms to recruit medical students for this survey introduces several notable limitations. First, the inherent selection bias associated with social media recruitment cannot be overlooked. As this method predominantly reaches individuals who are active on specific platforms, it may exclude potential respondents who do not use these platforms regularly or at all, potentially skewing the demographic and psychographic profile of the survey sample. Furthermore, the data collection process was restricted by the design of the survey tool, which did not allow respondents to save their progress and return to complete the survey at a later time. This limitation could have resulted in a higher dropout rate, particularly affecting those respondents who might have needed more time to reflect on their answers or who were interrupted during the process. In addition, the reliance on social media may also lead to a non-response bias, where the individuals choosing to participate may differ significantly in attitudes or behaviors from those who do not see or choose to ignore the survey invitation. These factors collectively contribute to the potential limitations in the generalizability and reliability of the study findings.

Implications for medical education, policy, and practice

These findings call for urgent attention from US medical education institutions. There is a need for more robust policies and educational programs that address sexual harassment and violence. Implementing comprehensive training programs for students and staff, establishing clear reporting mechanisms, and fostering a culture of zero tolerance toward sexual misconduct are imperative. Special emphasis should be placed on protecting vulnerable groups, such as non-heterosexual students and those identifying with non-traditional gender roles.

The study's results have implications for medical education policy and practice. There is a need for comprehensive strategies to prevent and address sexual misconduct. This includes developing robust training programs, establishing effective reporting mechanisms, and fostering a culture that actively discourages sexual harassment and violence. Special attention should be given to protecting and supporting vulnerable student populations.

Comparison with previous studies

These findings not only reaffirm prior research but also underscore the particularly disturbing prevalence of sexual misconduct and harassment among students, medical professionals, and faculty within higher education settings, highlighting an urgent need for systemic changes to address these pervasive issues [30-35]. However, this study provides more detailed insights into the specific experiences of medical students, a group often underrepresented in such research. The association between gender, sexual orientation, and the occurrence of unwanted sexual contact adds to the growing body of evidence on the vulnerabilities of minority groups in educational settings.

Importance of this study

This study helps to address a gap in the literature on sexual misconduct, harassment, and sexual violence in US undergraduate medical education. Notably, our research includes osteopathic medical students, a group often underrepresented in such studies. The inclusion of this demographic is significant, as it provides a more comprehensive understanding of the experiences of medical students in various educational contexts. By encompassing both allopathic and osteopathic students, this study helps to contribute to a more inclusive and complete picture of the prevalence, characteristics, and impacts of sexual misconduct in the medical academic community. This broader perspective is essential for developing targeted interventions and informing policies that cater to the diverse needs of all medical students.

Directions for future research

Future research should focus on understanding the underlying factors that contribute to the prevalence of sexual misconduct, harassment, and sexual violence in medical schools and the impact of such experiences on students' academic and personal well-being. In addition, exploring the effectiveness of current policies and training programs in preventing and addressing sexual harassment and violence can inform better practices and strategies in medical education environments.

Future research should also include longitudinal studies to track changes over time in the prevalence and nature of sexual harassment and violence in medical education. In addition, exploring the effectiveness of institutional policies and training programs in preventing and addressing these issues will be crucial. Research should also aim to understand the broader impacts of sexual misconduct on students' mental health, academic performance, and professional development. Lastly, the possible role of OMT in the facilitation of sexual misconduct in osteopathic medical schools should be investigated.

## Conclusions

This study reveals a troubling prevalence of sexual harassment and nonconsensual sexual contact among participants in this study, disproportionately affecting non-heterosexual and female students. The findings emphasize the need for medical schools to implement robust policies and programs to address and prevent sexual misconduct. Targeted interventions should focus on creating an inclusive environment that ensures the safety and well-being of all students, particularly those from vulnerable groups. In addition, the study underscores the importance of addressing power dynamics within medical education that may contribute to such incidents. Comprehensive and continuous efforts are required to foster a culture of respect and accountability, ultimately enhancing the educational experience and professional development of future healthcare practitioners.

## Appendices

Exploring Sexual Violence and Harassment in U.S. Medical Schools: A Cross-Sectional Study

INFORMED CONSENT University of the Incarnate Word School of Osteopathic Medicine TITLE OF STUDY: EXPLORING SEXUAL VIOLENCE AND HARASSMENT IN U.S. MEDICAL SCHOOLS: A CROSS-SECTIONAL STUDY INVESTIGATOR(S): Daryl Traylor, PhD, M.S., MPH, OMS-1, University of the Incarnate Word School of Osteopathic Medicine Eboni Anderson, DHEd, M.A., MEd, MSW, PhD (c), A.T. Still University School of Osteopathic Medicine in Arizona Taranjit Kaur, B.S., OMS-2, William Carey School of Osteopathic Medicine Harnoor Gill, B.S., OMS-2, William Carey School of Osteopathic Medicine Cooper Allenbrand, B.S., B.A., OMS-2, University of the Incarnate Word School of Osteopathic Medicine For questions or concerns about the research study, you may contact Daryl Traylor at 623-295-9081 or via email dtraylor@student.uiwtx.edu. You may also contact our faculty supervisor Dr. Linda Grace-Solis at lgsolis1@uiwtx.edu should you have any questions or concerns about the study.     

PURPOSE OF THIS STUDY: The purpose of this research study is to address the lack of comprehensive quantitative research on sexual harassment and sexual violence within U.S. medical schools. By quantifying the prevalence and identifying the factors associated with these incidents, this research study aims to provide an evidence-based understanding of the problem. The findings may contribute to the development of targeted interventions and policies that promote a safer and more inclusive educational environment for medical students. The research study survey instrument will ask about any instances of sexual harassment and/or sexual violence that you may have experienced while attending medical school. PARTICIPANTS: You are being asked to participate in this research study because you fit these criteria: You are 18 years of age or older, you can understand English, and are currently enrolled and attending a U.S. allopathic (MD) or osteopathic (DO) medical school. PROCEDURE: If you choose to participate in this research study, you will be asked to complete an anonymous survey delivered online via Qualtrics. BENEFITS OF PARTICIPATION: While participation in this research study will not present a direct benefit to you as an individual, your involvement may directly contribute to the creation of a safer educational environment. The findings from this research will help identify the factors associated with sexual harassment and sexual violence, enabling the development of effective interventions and policies. By participating, you become an integral part of fostering positive change within medical schools, ensuring that future generations of students can learn and thrive in an atmosphere free from harassment and violence.    

Secondly, your participation in this research study will raise awareness about the prevalence of sexual harassment and sexual violence in U.S. medical schools. By sharing your experiences and insights, you will help initiate important conversations that challenge the status quo and encourage institutions to take proactive measures to address these issues. Your involvement will play a vital role in fostering a culture of accountability and support within the medical community.    

Moreover, your contribution to this research has the potential to bring about long-term benefits for future medical students. The insights gained from this study may shape future policies, protocols, and educational programs aimed at preventing and addressing sexual harassment and sexual violence. By participating, you become an agent of change, empowering future generations of medical students to navigate their educational journey in a more respectful and supportive environment.    

We want to assure you that your participation will be treated with the utmost confidentiality. Your privacy will be protected, and your identity will remain anonymous throughout the study. The research team is committed to adhering to ethical guidelines to ensure your rights and well-being are safeguarded.    

RISKS OF PARTICIPATION: Personal questions are asked, which may make you feel uncomfortable. The study survey instrument will ask about any instances of sexual harassment and/or sexual violence that you may have experienced while attending medical school. The risks associated with this study are primarily psychological, including potential emotional distress or discomfort when recalling instances of sexual harassment and violence. These risks are anticipated to be infrequent, occurring in a small percentage of participants, and of low to moderate severity. However, the survey does not ask you to provide identifying information such as school attending, Internet Protocol address, name, address, email address, or phone number. Your answers will not be readily linked to you. In addition, all results will be reported as a group. Participants will be provided with resources and contact information for counseling services and support organizations specializing in sexual harassment and sexual violence. This will ensure that participants have access to support systems in the event that they experience any distress during or after participation in this study.     If you or someone you know is in need of resources regarding sexual misconduct, there are several confidential resources where students can get support. The links below can help you connect with those resources for you or someone you know.     

Confidential Resources:   Rape, Abuse, Incest Nation Network [RAINN] Hotline and Online Chat: https://www.rainn.org/resources   National Domestic Violence Hotline and online chat: https://www.thehotline.org/   National Violence and Sexual Resource Center: https://www.nsvrc.org/find-help     

COST / COMPENSATION: There will be no financial cost or compensation provided to you to participate in this study. The study will take about 10-15 minutes of your time. CONFIDENTIALITY:  All information gathered in this study will be kept confidential and anonymous. As stated earlier, no reference will be made in written or oral materials that could link you to this study. All records will be stored on a password protected, encrypted hard drive for 3 years after completion of the study. After the storage time, the information gathered will be destroyed as appropriate by the Principal Investigator. VOLUNTARY PARTICIPATION: Your participation in this study is voluntary. You may refuse to participate in this study or in any part of this study. You may withdraw at any time. You are encouraged to ask questions about this study at the beginning or any time during the research study. You may contact the University of the Incarnate Word Institutional Review Board (IRB) if you: Have any questions about your rights as a study participant            

Want to report any problems or complaints   

Feel under any pressure to take part or stay in this study     

The IRB is a group of people who review research studies to make sure the rights of participants are protected. Their phone number is (210) 805-3565 and the email address is irb@uiwtx.edu. 

Q1 Do you consent to participate in this study? 
 

o Yes  (1) 

o No  (2) 

Q2 Are you 18 years of age or older?
 

o Yes  (1) 

o No  (2) 

Q3 Are you currently attending a U.S. allopathic (MD) or osteopathic (DO) medical school?
 

o Yes  (1) 

o No  (2) 

Q4 Do you understand English?
 

o Yes  (1) 

o No  (2) 

Demographic Questions

Q1 What is your gender identity?

o Male  (1) 

o Female  (2) 

o Transgender male  (3) 

o Transgender female  (4) 

o Genderqueer/Gender‐nonconforming  (5) 

o Other  (6) 

Q2 What is your ethnicity?

o Hispanic or Latino  (1) 

o Not Hispanic or Latino  (2) 

Q3 What is your race? (mark all that apply)

▢        American Indian or Alaska Native  (1) 

▢        Asian  (2)

▢        Black or African American  (3) 

▢        Native Hawaiian or Other Pacific Islander  (4) 

▢        White  (5)

Q4 Which term best describes your sexual orientation?

o Bisexual  (1) 

o Gay  (2) 

o Heterosexual  (3) 

o Lesbian  (4) 

o Questioning  (5) 

o Other  (6) 

Q5 What is your current status in medical school?

o 1st year  (1) 

o 2nd year  (2) 

o 3rd year  (3) 

o 4th year  (4) 

Q6 What type of medical school do you attend?

o Allopathic (MD granting)  (1) 

o Osteopathic (DO granting)  (2) 

Q7 How old are you? ________________________________________________________________

Perceptions of campus leadership, policies, and reporting

Q8 If someone were to report a sexual assault to a campus authority, how likely is it that:

Very Likely (1)

Moderately Likely (2)

Slightly Likely (3)

Not at all Likely (4)

**Table 9:** Perceptions of campus leadership, policies, and reporting

	Very Likely (1)	Moderately Likely (2)	Slightly Likely (3)	Not at all Likely (4)
The university would take the report seriously. (1)				
The University would keep the report private (2)				
The university would forward the report outside the campus to criminal investigators. (3)				
The university would take steps to protect the safety of the person making the report. (4)				
The university would support the person making the report. (5)				
The university would take corrective action to address factors that may have led to the sexual assualt (6)				
The university would take corrective action against the offender. (7)				
The university would take steps to protect the person making the report from retaliation. (8)				
Students, faculty, and staff would label the person making the report as a troublemaker. (9)				
Students, faculty, and staff would support the person making the report. (10)				
The alleged offender(s) or their associates would retaliate against the person making the report. (11)				
My education and career might suffer if I make a report. (12)				

Q9 Have you received training in policies and procedures regarding incidents of sexual assault (e.g. what is defined as sexual assault, how to report an incident, confidential resources, procedures for investigating)?

o Yes  (1) 

o No  (2) 

Q10 Have you received training in prevention of sexual assault?

o Yes  (1) 

o No  (2) 

Q11 If yes, how useful did you think the training was?

o Not at all useful  (1) 

o Slightly useful  (2) 

o Moderately useful  (3) 

o Very useful  (4) 

o Extremely useful  (5) 

Q12 Please indicate your level of agreement to the following statements:

Strongly agree (1)

Somewhat agree (2)

Neither agree nor disagree (3)

Somewhat disagree (4)

Strongly disagree (5)

**Table 10:** Perceptions of campus leadership, policies, and reporting

	Strongly agree (1)	Somewhat agree (2)	Neither agree nor disagree (3)	Somewhat disagree (4)	Strongly disagree (5)
If a friend or I were sexually assaulted, I know where to go to get help. (1)					
I understand my university's formal process to address sexual assault complaints. (2)					
I have confidence that my university administers the formal process to address sexual assault complaints fairly. (3)					

This section asks about nonconsensual or unwanted sexual contact you may have experienced. When you are asked about whether something happened since you have been in medical school, please think about what has happened since 2020 The person (s) with whom you had the unwanted sexual contact could have been a stranger or someone you know, such as a classmate/peer, faculty, or staff member. These questions ask about five types of unwanted sexual contact: a. forced touching of a sexual nature (forced kissing, touching of private parts, grabbing, fondling, rubbing up against you in a sexual way, even if it is over your clothes) b. oral sex (someone’s mouth or tongue making contact with your genitals or your mouth or tongue making contact with someone else’s genitals) c. sexual intercourse (someone’s penis being put in your vagina) d. anal sex (someone’s penis being put in your anus) e. sexual penetration with a finger or object (someone putting their finger or an object like a bottle or a candle in your vagina or anus) If you or someone you know is in need of resources regarding sexual misconduct, there are a number of confidential resources where students can get support. The links below can help you connect with those resources for you or someone you know. Confidential Resources: Rape, Abuse, Incest Nation Network [RAINN] Hotline and Online Chat: https://www.rainn.org/resources National Domestic Violence Hotline and online chat: https://www.thehotline.org/ National Violence and Sexual Resource Center: https://www.nsvrc.org/find-help

Q13 Has anyone, since 2020, had sexual contact with you by using physical force or threatening to physically harm you?

o Yes  (1) 

o No  (2) 

Q14 Has anyone, since 2020, attempted but not succeeded in having sexual contact with you by using or threatening to use physical force against you?

o Yes  (1) 

o No  (2) 

Q15 Since 2020, has anyone had sexual contact with you when you were unable to provide consent or stop what was happening because you were passed out, drugged, drunk, incapacitated, or asleep? This question asks about incidents that you are certain happened.

o Yes  (1) 

o No  (2) 

Q16 Since 2020, have you suspected that someone has had sexual contact with you when you were unable to provide consent or stop what was happening because you were passed out, drugged, drunk, incapacitated, or asleep? This question asks about events that you think (but are not certain) happened.

o Yes  (1) 

o No  (2) 

Q17 When the person(s) had sexual contact with you when you were unable to provide consent or stop what was happening because you were passed out, drugged, drunk, incapacitated, or asleep, which of the following happened? Please check all that apply.

▢        Forced touching of a sexual nature  (1) 

▢        Oral sex  (2) 

▢        Sexual intercourse  (3) 

▢        Anal sex  (4) 

▢        Sexual penetration with a finger or object  (5) 

▢        I don't know  (6) 

▢        N/A  (7) 

Q18 Who did the UNWANTED BEHAVIOR involve? (You may check more than one)

▢        Another student or peer  (1) 

▢        Faculty at your medical school  (2) 

▢        Staff member at your medical school  (3) 

▢        A resident physician  (4) 

▢        An attending physician  (5) 

▢        Someone else affiliated with your medical school or hospital/clinic where you are rotating  (6) 

▢        Other  (8)

▢        N/A  (9) 

Q19 What was the gender of the individual who did this to you?

o Male  (1) 

o Female  (2) 

o Non-binary  (3) 

o Not sure  (4) 

o N/A  (5) 

Q20 Where did the incident occur? (Mark ALL that apply)

▢        Off campus  (1) 

▢        On campus  (2) 

▢        A clinical site  (3) 

▢        Other location  (4) 

▢        N/A  (5) 

Q21 Did you initiate the university's process to file a sexual assault complaint?
 

o Yes  (1) 

o No  (2) 

o NA  (4) 

Q22 If yes, did university's formal procedures help you deal with the problem?

o Didn't help me at all  (1) 

o Helped me a little  (2) 

o Helped, but could have helped more  (3) 

o Helped me a lot  (4) 

o Completely solved the problem  (5) 

o N/A  (6) 

Q23 If you did not tell anyone, why? (Select ALL that apply)

▢        Ashamed/embarrassed  (1) 

▢        Fear of retribution from the person who did it  (2) 

▢        Fear of not being believed by my school  (3) 

▢        Didn't know my school's reporting procedure  (4) 

▢        I did not feel that my campus leadership would solve the problem  (5) 

▢        Feared that reporting the incident would impact my academics  (6) 

▢        Feared that reporting the incident would impact my chances of earning the residency that I want  (7) 

▢        Didn’t want others to worry about me  (8) 

▢        Didn't think that my school would do anything about the report  (9) 

▢        Wanted to forget it happened  (10) 

▢        Didn’t want others to worry about me  (11) 

▢        N/A  (12) 

The next section asks about your experiences with sexual harassment since starting medical school. Sexual harassment encompasses various types of unwelcome behavior, whether expressed verbally, non-verbally, or physically, that is of a sexual nature and results in an environment that is intimidating, hostile, degrading, or offensive. Examples of such behavior include physical contact, encroachment on personal space, suggestive remarks, unsolicited comments on appearance or attire, sexually-oriented jokes, or the exhibition of sexually offensive material in a public setting. If you or someone you know is in need of resources regarding sexual misconduct, there are a number of confidential resources where students can get support. The links below can help you connect with those resources for you or someone you know. Confidential Resources: Rape, Abuse, Incest Nation Network [RAINN] Hotline and Online Chat: https://www.rainn.org/resources National Domestic Violence Hotline and online chat: https://www.thehotline.org/ National Violence and Sexual Resource Center: https://www.nsvrc.org/find-help ________________________________________________________________

Q24 Have you ever experienced any episodes of sexual harassment since 2020?

o Yes  (1) 

o No  (2) 

Q25 How often has this happened to you since 2020?
 

o Daily  (1) 

o Weekly  (2) 

o Monthly  (3) 

o Only once  (4) 

o 2-4 times  (5) 

o 5-10 times  (6) 

o More than 10 times  (7) 

o N/A  (8) 

Q26 What type(s) of sexual harassment have you experienced since 2020? (CHECK ALL THAT APPLY)
 

▢        Unwanted physical contact  (1) 

▢        Suggestive remarks  (2) 

▢        Jokes of a sexual nature  (3) 

▢        Display of sexually offensive materials in a public space  (4) 

▢        Unwanted comments on dress or appearance  (5) 

▢        Invasion of personal space  (6) 

▢        Staring or Leering  (7) 

▢        Intimidating presence (e.g following at close proximity)  (8) 

▢        None  (9) 

Q27 Where did this happen to you? (SELECT ALL THAT APPLY)
 

▢        On campus  (1) 

▢        Hospital or clinic  (2) 

▢        Other  (3)

▢        N/A  (4) 

Q28 Who did this to you? (Select all that apply)
 

▢        Student or peer  (1) 

▢        Faculty member  (2) 

▢        Staff member on campus  (3) 

▢        Resident physician  (4) 

▢        Attending physician  (5) 

▢        Staff member at hospital or clinic  (6) 

▢        Other  (7)

▢        N/A  (8) 

Q29 Did you feel you needed support or help from your school with this?

o Yes  (1) 

o No  (2) 

o NA  (4) 

Q30 If yes, did you get the support you needed from your school?

o Yes  (1) 

o No  (2) 

o NA  (4) 

You have reached the end of the survey.  We really appreciate your honesty and for taking the time to share your experiences with us. We understand that this may have been difficult for you, but we value your responses, and they will help us improve the campus climate for current and future U.S. medical students.  If you or someone you know is in need of resources regarding sexual misconduct, there are a number of confidential resources where students can get support. The links below can help you connect with those resources for you or someone you know. Confidential Resources: Rape, Abuse, Incest Nation Network [RAINN] Hotline and Online Chat: https://www.rainn.org/resources National Domestic Violence Hotline and online chat: https://www.thehotline.org/ National Violence and Sexual Resource Center: https://www.nsvrc.org/find-help
 
